# Estimating the basic reproduction number for single-strain dengue fever epidemics

**DOI:** 10.1186/2049-9957-3-12

**Published:** 2014-04-07

**Authors:** Adnan Khan, Muhammad Hassan, Mudassar Imran

**Affiliations:** 1 Department of Mathematics, Lahore University of Management Sciences, DHA, Lahore, Pakistan; 2 Department of Mathematics, Swiss Federal Institute of Technology (ETH), Zurich, Rämistrasse 101, 8092 Zurich, Switzerland

**Keywords:** Epidemiology, Dengue fever, Statistical inference, Stochastic model, Markov chain Monte Carlo

## Abstract

**Background:**

Dengue, an infectious tropical disease, has recently emerged as one of the most important mosquito-borne viral diseases in the world. We perform a retrospective analysis of the 2011 dengue fever epidemic in Pakistan in order to assess the transmissibility of the disease. We obtain estimates of the basic reproduction number *R*_0 _from epidemic data using different methodologies applied to different epidemic models in order to evaluate the robustness of our estimate.

**Results:**

We first estimate model parameters by fitting a deterministic ODE vector-host model for the transmission dynamics of single-strain dengue to the epidemic data, using both a basic ordinary least squares (OLS) as well as a generalized least squares (GLS) scheme. Moreover, we perform the same analysis for a direct-transmission ODE model, thereby allowing us to compare our results across different models. In addition, we formulate a direct-transmission stochastic model for the transmission dynamics of dengue and obtain parameter estimates for the stochastic model using Markov chain Monte Carlo (MCMC) methods. In each of the cases we have considered, the estimate for the basic reproduction number *R*_0 _is initially greater than unity leading to an epidemic outbreak. However, control measures implemented several weeks after the initial outbreak successfully reduce *R*_0 _to less than unity, thus resulting in disease elimination. Furthermore, it is observed that there is strong agreement in our estimates for the pre-control value of *R*_0_, both across different methodologies as well across different models. However, there are also significant differences between our estimates for the post-control value of the basic reproduction number across the two different models.

**Conclusion:**

In conclusion, we have obtained robust estimates for the value of the basic reproduction number *R*_0 _associated with the 2011 dengue fever epidemic before the implementation of public health control measures. Furthermore, we have shown that there is close agreement between our estimates for the post-control value of *R*_0 _across the different methodologies. Nevertheless, there are also significant differences between the estimates for the post-control value of *R*_0 _across the two different models.

## Multilingual abstracts

Please see Additional file 1 for translations of the abstract into the six official working languages of the United Nations.

## Background

Global incidence of dengue has seen a striking increase over the past few decades [1,2]. The infectious disease is now endemic in more than a hundred tropical and subtropical countries worldwide [1-3]. With an estimated 50–100 million cases and nearly 10,000- 20,000 deaths annually, dengue ranks second to Malaria amongst deadly mosquito-borne diseases [1,2,4-6]. The disease is caused by one of four virus serotypes (strains) of the genus *Flavivirus*[2,3,7]. Most infected individuals suffer from dengue fever, a severe flu-like illness characterized by high fever, which is not usually a threat to mortality [2,8]. The symptoms of the disease last one to two weeks, after an initial incubation period of about 4–7 days [9]. Some infected individuals however, develop dengue hemorrhagic fever (DHF) resulting in bleeding, low levels of blood platelets and blood plasma leakage, or dengue shock syndrome (DSS) resulting in extremely low blood pressures. The risk associated with DHF and DSS is considerably higher, with mortality ranging from 5–15% [3,5,9,10].

There is evidence of dengue epidemics occurring in North America, Asia and Africa in the late 18^*th*^ century [3]. Up until the middle of the 20^*th*^ century however, incidences of dengue fever have been rare [3]. Nonetheless, since the 1970’s, there has been a marked increase in the number of dengue cases, as well as the frequency and severity dengue epidemics, with the WHO claiming a 30-fold increase in the incidence of dengue between 1960 and 2010 [2,3,8]. Factors such as population growth, rapid urbanization and increase international travel are often cited as having contributed to this dramatic increase [8]. Dengue is currently endemic in nearly 110 countries in Southeast Asia, the Americas, Africa and the Eastern Mediterranean [2]. The WHO estimates that nearly 2.5 billion people are at risk of contracting the disease. Furthermore, nearly 50–100 million cases and almost 20,000 deaths due to more severe forms of dengue fever are reported globally every year, making dengue one of the deadliest mosquito-transmitted diseases [1,2,4-6].

Dengue is transmitted to humans through mosquito bites. Female mosquitos of the *Aedas* genus, primarily *Aedes aegypti*, acquire the dengue virus through a blood meal from infected humans [2,11]. The dengue virus has an incubation period of about 7–10 days in the vector, and is then spread to susceptible humans who are bitten by the infected mosquito [9]. The virus also has an incubation period of 4–7 days in the host [9]. While vectors never recover from infection with the dengue virus, the infection in hosts lasts only about one to two weeks [2]. Hosts that recover from infection with one serotype of the dengue virus gain life-long immunity from that serotype but only temporary and partial immunity to other serotypes [2,4,12-14]. This partial cross-immunity is the cause of antibody-dependent enhancement (ADE) in the setting of a secondary infection with a different serotype of DENV (Dengue Virus). ADE is hypothesized to be one factors leading to DHF and DSS, the more severe form of dengue disease [4,12,13,15]. In this study however, we will consider infection involving only a single serotype of the dengue virus.

About 80% of individuals suffering from a primary infection with DENV are asymptomatic or display only a mild, uncomplicated fever [2,8]. A much smaller proportion of infected individuals suffer from dengue hemorrhagic fever and dengue shock syndrome [3,5,9]. As mentioned previously however, risk of DHF and DSS is associated primarily with secondary infection with a heterologous serotype of DENV [4,12,13,15]. In general, the course of infection of dengue can be divided into three separate phases: febrile, critical and recovery. The febrile phase, which is rarely life threatening, is marked by the sudden onset of high fever, rash, headaches and muscle and joint pains, which lead to the alternative name "breakbone fever" for dengue disease [8]. While most individuals then progress to the recovery phase, a small fraction of infected individuals instead progress to the critical phase of the disease. This phase lasts for one or two days and is marked by low blood pressure, leakage of blood plasma from the capillaries and decreased blood supply to organs. Severe cases of these symptoms are associated with DHF and DSS and the mortality in this phase of the disease is estimated to be as high as 5–15% [3,5,8,9].

Over the past several years, a number of deterministic mathematical models have been proposed to analyze the transmission dynamics of dengue in urban communities [5,11-17]. L. Esteva and C. Vargas [14] have investigated the coexistence of two serotypes of dengue virus using a deterministic ODE model. Moreover, Ferguson et al. [15] have investigated the effects of ADE on the transmission of multiple serotypes of dengue virus. In addition, Garba et al. [11] have shown the existence of a backward bifurcation in a standard incidence ODE model for a single strain of dengue virus. Garba et al. [12] have also explored the effects of cross-immunity on the transmission dynamics of two strains of dengue virus. Similarly, H. Wearing and P. Rohani [13] have investigated the effects of both ADE and cross immunity on multiple serotypes of dengue virus. Finally, Chowell et al. [18] have estimated the basic reproduction number for dengue using spatial epidemic data.

In addition, over the past few decades, several stochastic epidemic models for the spread of infectious diseases have also been proposed and analyzed [19-27]. An important qualitative difference between deterministic and stochastic epidemic models in general is the asymptotic dynamics [28]. Furthermore, stochastic models also allow for the possibility of disease extinction in finite time and therefore the expected time to disease extinction can be calculated [19,28,29]. It is also observed that stochastic models better capture the uncertainty and variability that is inherent in real-life epidemics due to factors such as the unpredictability of person-to-person contact [27,29]. L. J. S. Allen [28,29] has explored the utility of stochastic epidemic models by comparing them with deterministic models. Despite, the utility of stochastic models, however, very little stochastic modeling has been performed for the transmission dynamics of dengue virus (see [26] and the references therein).

The purpose of this study is to estimate the transmissibility of the dengue virus during the 2011 dengue fever epidemic in Pakistan using epidemic data in the form of the cumulative number of reported cases of dengue. We will employ three different techniques, applied to two different models and compare the results across both the different statistical inference methodologies as well as the different models. The first approach, based on the earlier work of Cintron-Arias et al. [30], will involve fitting a deterministic epidemic model for the transmission of dengue to the epidemic data using an ordinary least squares (OLS) scheme implemented using the built-in optimization toolbox in MATLAB and applied in the context of an appropriate statistical model. The second method, also based on the recent work of Cintron-Arias et al. [30], will use a generalized least squares (GLS) scheme to fit the same deterministic model to the epidemic data. Furthermore, both approaches will also be applied to a different direct-transmission model for the transmission dynamics of dengue. Finally, the third approach will involve the formulation of a direct transmission stochastic epidemic model for dengue. We shall then use Markov chain Monte Carlo techniques to obtain a probability distribution for the model parameters.

A simple but effective measure of the transmissibility of an infectious disease is given by the basic reproduction number *R*_0_, defined as the total number of secondary infections produced by introducing a single infective in a completely susceptible population [31]. For vector-borne diseases such as malaria and dengue, *R*_0_ is the number of secondary cases produced by a single infectious vector introduced in a completely susceptible host and vector population. In general, for simple epidemic models, if *R*_0 _is greater than unity, an epidemic will occur while if *R*_0 _is less than unity, an outbreak will most likely not occur. Thus, the value of *R*_0 _can be used to determine the intensity of control measures that need to be implemented in order to contain the epidemic.

The estimation of the basic reproductive number is generally an indirect process because the model parameters that *R*_0 _depends on are difficult or impossible to determine directly. The general methodology used therefore, attempts to fit an epidemic model to available epidemic data in order to estimate the model parameters. These parameters are then used to estimate the basic reproduction number *R*_0_. The current study is based on this methodology.

## Results

**Figure 1:**
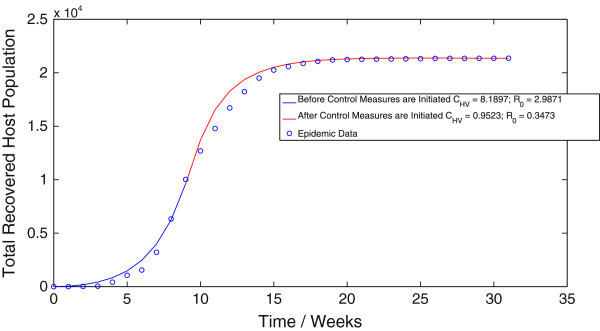
**The best-fit trajectory of model (**1.1**) calculated using the OLS methodology, along with the epidemic data.**

**Figure 2:**
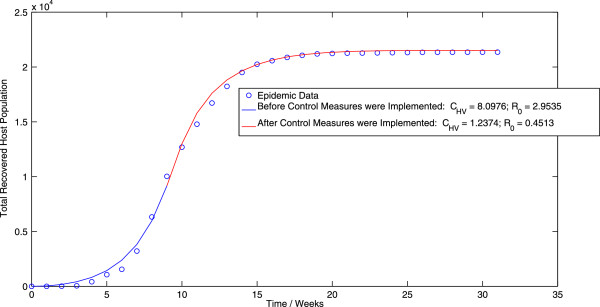
**The best-fit trajectory of model (**1.1**) calculated using the GLS methodology, along with the epidemic data.**

Applying the algorithm for the ordinary least squares (OLS) methodology to the vector-host model (1.1) results in a value of *C*_*HV *_= 8.1897 week ^-^1 before the implementation of control measures and a value of *C*_*HV *_= 0.9523 week ^-^1 after the implementation of control measures. Thus, we obtain an estimate of *R*_0 _= 2.9871 before the implementation of control measures and *R*_0 _= 0.3473 after the implementation of control measures. The best-fit trajectory of model (1.1) calculated using the OLS methodology, along with the epidemic data is displayed in Figure 1. Similarly, implementing the algorithm for the generalized least squares (GLS) methodology results in a value of *C*_*HV *_= 8.0976 week ^-1 ^before the implementation of control measures and a value of *C*_*HV *_= 1.2374 week ^-1^ after the implementation of control measures. Thus, when using the GLS scheme, we obtain an estimate of *R*_0 _= 2.9535 before the implementation of control measures and *R*_0 _= 0.4513 after the implementation of control measures. Figure 2 displays the best-fit trajectory of model (1.1) calculated using the GLS methodology. We observe that the GLS estimates for *R*_0 _before the implementation of control measures are in close agreement with the results obtained using the OLS scheme. There is however, difference between the estimates for *R*_0 _after the implementation of control measures.

**Figure 3:**
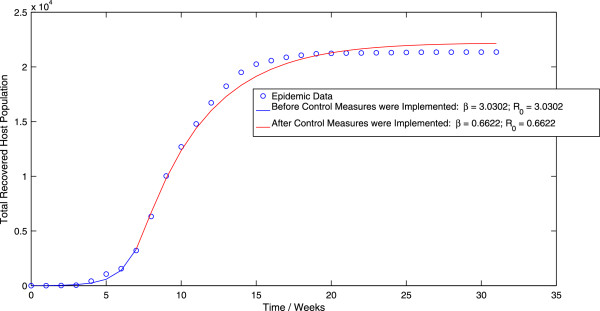
**The best-fit trajectory of model (**3.1**) calculated using the OLS methodology, along with the epidemic data.**

Application of the algorithm for the ordinary least squares (OLS) methodology to the direct-transmission model (3.1) results in a value of *β *= 3.0302 week ^-1^ before the implementation of control measures and a value of *β*=0.6622 week ^-1^ after the implementation of control measures. This corresponds to a value of *R*_0 _= 3.0182 before the implementation of control measures and *R*_0 _= 0.6596 after the implementation of control measures. Figure 3 displays the best-fit trajectory of the direct-transmission model (3.1) calculated using the OLS scheme. We observe that the pre-control value of *R*_0 _is slightly larger (1.04%) than the corresponding value obtained by applying the OLS scheme to the vector-host model (1.1), which we feel is not a statistically significant increase. However, the post-control value of *R*_0 _is significantly higher (89.92%) than the corresponding value obtained by applying the OLS scheme to the vector-host model (1.1).

**Figure 4:**
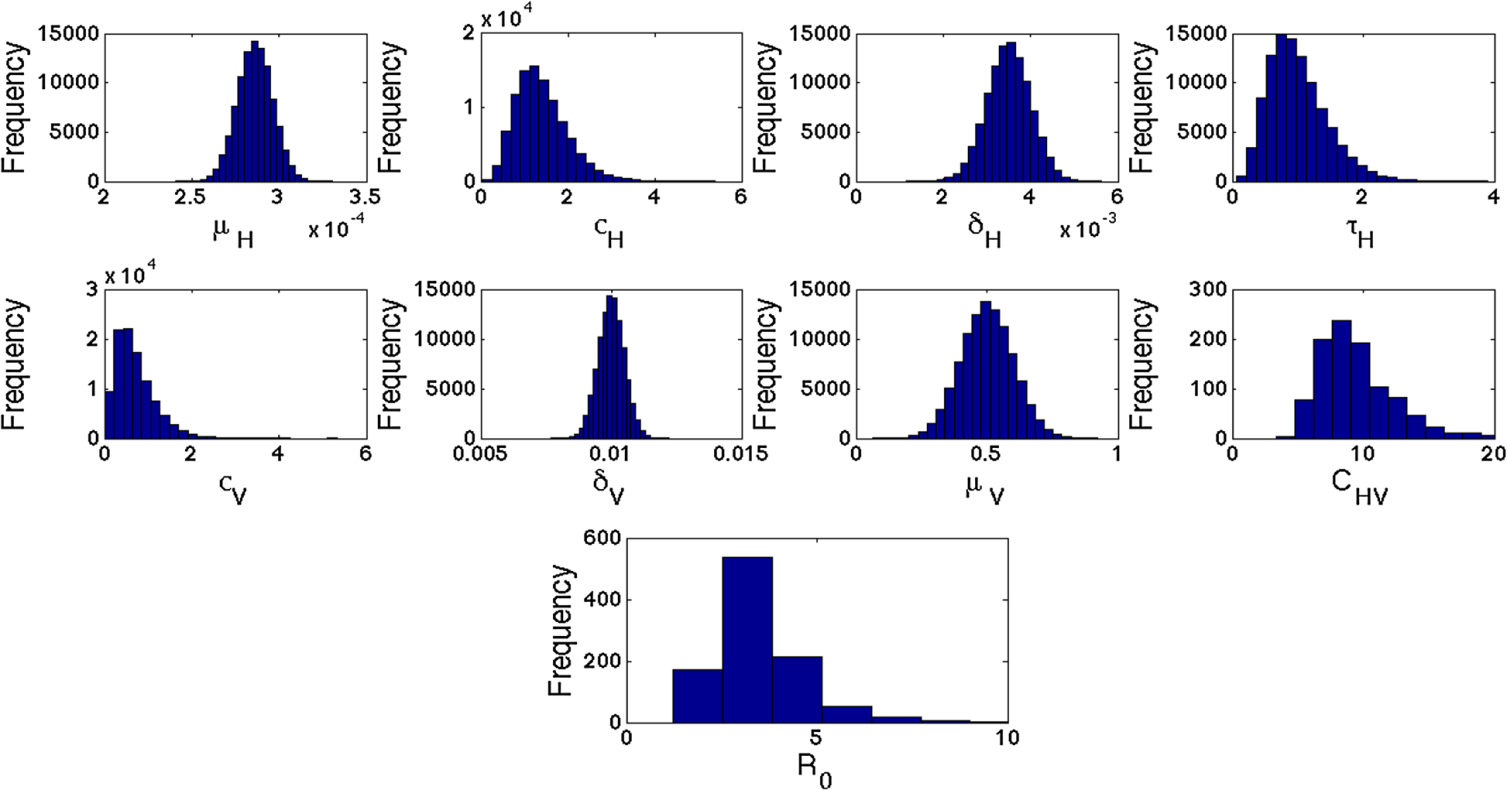
**Uncertainty analysis of the basic reproduction number** ***R***_**0**_**.**

**Figure 5:**
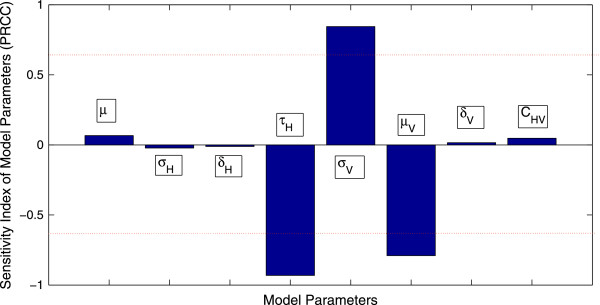
**Sensitivity Analysis of the Basic Reproduction Number** ***R***_**0**_**.**

In addition, pre-control estimates of *R*_0 _for the dengue epidemic from the uncertainty analysis are shown in Figure 4. The 95% confidence interval for the pre-control value of *R*_0 _is given by (2.0293,6.5310). The probability that *R*_0_, before the implementation of control measures, is greater than unity is more than 99.9%. Similarly, the sensitivity analysis, shown in Figure 5, suggests that the most significant (PRCC values above 0.5 or below -0.5) sensitivity parameters to *R*_0 _are *τ*_*H*_, *σ*_*V *_and *μ*_*V*_. This suggests that these parameters need to be estimated with precision in order to accurately capture the transmission dynamics of dengue.

**Figure 6:**
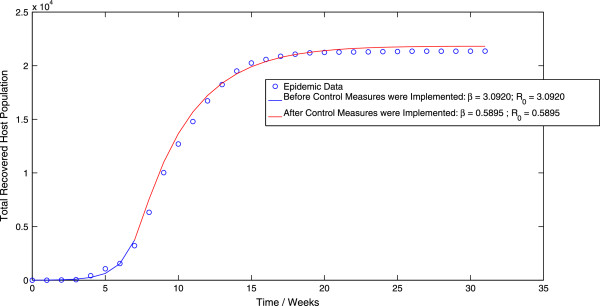
**The best-fit trajectory of model (**3.1**) calculated using the GLS methodology, along with the epidemic data.**

Furthermore, implementing the algorithm for the generalized least squares methodology in the case of the direct-transmission model (3.1) results in a value of *β *= 3.0920 week ^-1^ before the implementation of control measures and a value of *β *= 0.5895 week ^-1^ after the implementation of control measures. These values of the contact rate result in an estimate of *R*_0 _= 3.0797 before the implementation of control measures and *R*_0 _= 0.5872 after the implementation of control measures. The best-fit trajectory of the direct-transmission model (3.1) calculated using the GLS scheme is displayed in Figure 6. We observe that while these results are in close agreement with the corresponding results obtained by applying the OLS scheme to the direct-transmission SEIR model, the estimated value of post-control *R*_0 _is again, significantly larger (30.11%) than the corresponding value obtained by applying the GLS scheme to the vector-host model. However, there is only a slight increase (4.27%) in the estimate for the pre-control value of *R*_0 _across the different models, which we again feel is not a statistically significant increase.

**Figure 7:**
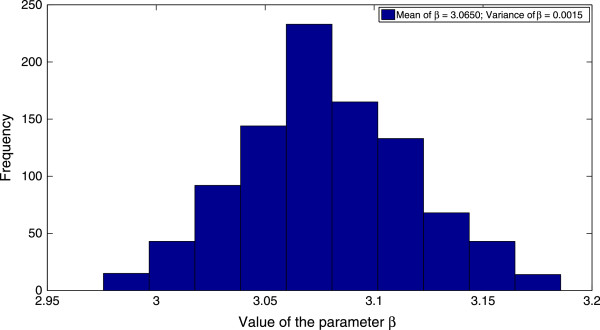
**The distribution of the parameter *β *** **before the implementation of control measures.**

**Figure 8:**
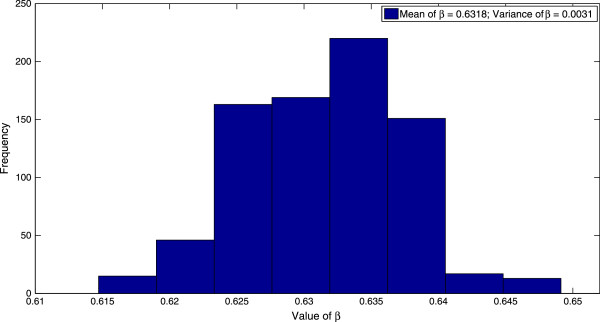
**The distribution of the parameter *β *** **after the implementation of control measures.**

**Figure 9:**
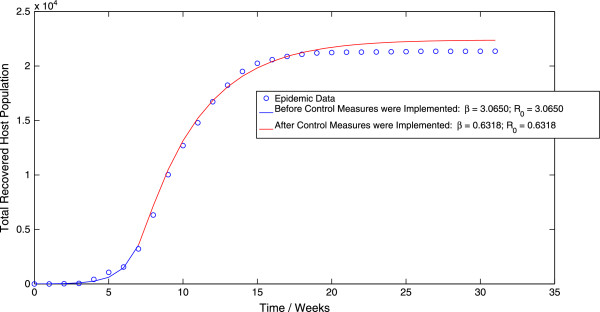
**The mean trajectory of the stochastic model (**4.1**), calculated using Monte Carlo simulations involving the mean of the posterior distribution, plotted along with the epidemic data.**

Finally, implementation of the MCMC algorithm to the stochastic direct-transmission model (4.1) results in a value of *β *= 3.0650 week ^-1^ before the implementation of control measures and a value of *β *= 0.6318 week ^-1^ after the implementation of control measures. These values of the contact rate correspond to values of *R*_0 _= 3.0528 before the implementation of control measures and *R*_0 _= 0.6293 after the implementation of control measures. The probability distributions of the contact rate *β*, both before and after the implementation of control measures are displayed in Figure 7 and Figure 8 respectively. Moreover, the mean trajectory of the stochastic model (4.1) calculated using Monte Carlo simulations involving the mean of the posterior distribution is displayed in Figure 9. The results of the MCMC methodology are in close agreement with the results obtained by applying the GLS and OLS schemes to the deterministic direct-transmission model (3.1). Furthermore, there is also close agreement in our estimates for the pre-control value of *R*_0 _across both the previous methodologies as well as the different models. There is however, again, a significant difference between the post-control value of *R*_0_, obtained using the MCMC algorithm and the estimates of the post-control value of *R*_0_, obtained by applying the OLS and GLS scheme to the vector-host model (1.1).

## Discussion

We have performed a retrospective analysis of the 2011 dengue fever epidemic in Pakistan and obtained estimates of the basic reproduction number *R*_0_, from epidemic data using three different methods. *R*_0_, defined as the total number of secondary infections produced by introducing a single infective in a completely susceptible population, is a simple but effective measure of the transmissibility of an infectious disease. In each case it was observed that the value of *R*_0 _was initially well in excess of unity, leading to the observed epidemic outbreak. Some weeks after the initial outbreak however, control measures were successfully implemented that reduced the value of *R*_0 _to less than unity, thus resulting in disease elimination.

Several methods have been proposed for the estimation of *R*_0_, both for deterministic as well as for stochastic models. These methods depend upon the mathematical model of the disease as well the nature of the data. In the case of deterministic compartmental models, least squares fit to the data has been widely used to estimate the model parameters [18,30,32]. For stochastic models likelihood based techniques have been used by several authors [33,34]. We consider two ODE based deterministic models and a Continuous Time Markov Chain (CTMC) based stochastic model. Using least squares estimation for the deterministic models and a Markov Chain Monte Carlo based approach for the stochastic model, we compare the value of *R*_0 _obtained using the different models. We note that this is the first such study performed for the Dengue Epidemic in Pakistan.

The first inference methodology involved fitting a deterministic ODE model for the transmission dynamics of single-strain dengue to the epidemic data using a basic ordinary least squares (OLS) scheme in the context of a statistical model which assumed longitudinally constant variance for the epidemic data. An a priori more realistic methodology was used to fit the deterministic ODE model to obtain estimates of the model parameters using a generalized least squares (GLS) scheme which made use of a statistical model that assumed that variances associated with the observation process were directly proportional to the measurement values. One of the questions we tried to address was whether or not the variances were strongly dependent on the observations. Finally, we formulated a discrete-time, direct transmission, stochastic model for the spread of dengue virus and used Markov chain Monte Carlo (MCMC) methods to perform Bayesian inference and estimate the basic reproduction number.

We observe that the estimates for the basic reproduction number *R*_0 _before the implementation of control measures are in excellent agreement for the same model across different methodologies. Similarly, across different models there is a very slight but nonetheless statistically insignificant difference in our estimates of the pre-control basic reproduction number. We therefore conclude that our estimation of the pre-control value of *R*_0 _is quite robust, both across different methodologies as well as across different models. This leads us to believe that the noise does not depend significantly on the data. Furthermore, agreement in our estimates across models indicates that both the vector-host model as well as the direct-transmission model can be used to accurately capture the disease dynamics of actual dengue epidemics before the implementation of control measures.

While there is also close agreement in our estimates for the basic reproduction number *R*_0 _after the implementation of control measures across different methodologies, there is nonetheless significant difference between the post-control estimates of *R*_0 _across the vector-host and direct-transmission models. Specifically, *R*_0 _is estimated to be significantly larger in value when using the direct-transmission model as opposed to the vector-host model. We conjecture that this might be due to the fact that the direct-transmission model makes use of an approximation, which involves solving for the equilibrium value of the vector force of infection. Thus, while the vector force of infection rises and peaks for the vector-host model, before settling to its equilibrium value, it is in effect equal to the smaller equilibrium value for the direct-transmission model. Therefore, since the vector force of infection for the direct transmission model is, in effect, smaller for the time period after the implementation of control measures, it results in a larger estimate of the basic reproduction number in order to produce a ‘best-fit’ for the observed epidemic data.

## Conclusion

In conclusion, we have attempted to assess the transmissibility of the dengue virus during the 2011 dengue fever epidemic in Pakistan by estimating the basic reproduction number *R*_0 _both before and after the implementation of public health control measures. Our estimates for the pre-control value of *R*_0 _are in close agreement both across different methodologies and the different models. Furthermore, the post-control estimates are also in close agreement across the different methodologies. There is however, a significant increase in the estimates of the post-control value of *R*_0 _obtained using the direct-transmission model compared to estimates obtained using the vector-host model.

## Methods

### Methods and materials for statistical inference using the vector-host model

#### ***The vector-host epidemic Model***

The model is a deterministic vector-host ODE model that assumes a homogeneous mixing of the host (human) and vector (mosquito) populations. The total human population at time *t*, denoted by *N*(*t*), is divided into four mutually exclusive classes comprising of susceptible humans *S*_*H*_(*t*), exposed humans *E*_*H*_(*t*), infected humans *I*_*H*_(*t*) and recovered humans *R*_*H*_(*t*). It is assumed that individuals who recover from infection with a particular serotype of Dengue gain lifelong immunity to it [2]. Similarly, the total vector population at time *t* is denoted by *N*_*V*_(*t*) and is divided into three mutually exclusive classes comprising susceptible of susceptible vectors *S*_*V*_(*t*), exposed vectors *E*_*V*_(*t*) and infected vectors *I*_*V*_(*t*). It is assumed that vectors (mosquitoes) infected with a particular serotype of Dengue never recover [2]. We also modify the original model of Garba et al. [11] by assuming that exposed humans and exposed vectors do not transmit the disease.

The model assumes that the susceptible human population *S*_*H*_(*t*) has a constant recruitment rate *Π*_*H *_and natural death rate *μ*. Susceptible individuals are infected with Dengue virus (due to contact with infected vectors) at a rate *λ*_*H *_and thus enter the exposed class *E*_*H*_. The exposed population *E*_*H*_(*t*) is depleted at the natural death rate *μ*. Additionally, exposed individuals develop symptoms and move into the infected class *I*_*H *_at a rate *σ*_*H*_. The infected population *I*_*H*_(*t*) is depleted via the natural death rate *μ*, the disease-induced death rate *δ*_*H *_and the recovery rate of infected individuals *τ*_*H*_. Finally, the recovered population *R*_*H*_(*t*) decreases due to the natural death rate *μ*.

Similarly, the susceptible vector population *S*_*V*_(*t*) has a constant recruitment rate *Π*_*V *_and a natural death rate *μ*_*V*_. Susceptible vectors are infected with Dengue virus (due to effective contact with infected humans) at a rate *λ*_*V *_and thus move to the exposed vector class *E*_*V*_. The exposed vector class *E*_*V*_(*t*) is depleted at the natural death rate *μ*_*V*_. In addition exposed vectors develop symptoms and move to the infected vector class *I*_*V *_at a rate *σ*_*V*_. Infected vectors, in addition to the natural death rate *μ* die at a disease induced death rate *δ*_*V*_.

Mathematically, the model is a follows

(1.1) $$\begin{array}{ll} \frac{dS_{H}}{\text{dt}} & =\Pi_{H}-\lambda_{H}S_{H}-\mu S\\ \frac{dE_{H}}{\text{dt}} & =\lambda_{H}S-(\sigma_{H}+\mu )E_{H}\\ \frac{dI_{H}}{\text{dt}} & =\sigma_{H}E_{H}-(\tau_{H}+\mu +\delta_{H})I_{H}\\ \frac{dR_{H}}{\text{dt}} & =\tau_{H}I_{H}-\mu R_{H}\\ \frac{dS_{V}}{\text{dt}} & =\Pi_{V}-\lambda_{V}S_{V}-\mu_{V}S_{V}\\ \frac{dE_{V}}{\text{dt}} & =\lambda_{V}S_{V}-(\sigma_{V}+\mu_{V})E_{V}\\ \frac{dI_{V}}{\text{dt}} & =\sigma_{V}E_{V}-(\mu_{V}+\delta_{V})I_{V}\\ \text{where,}\\ \lambda_{H} & =C_{\text{HV}}\frac{I_{V}}{N_{H}}\\ \lambda_{V} & =C_{\text{HV}}\frac{I_{H}}{N_{H}} \end{array}$$

A description of the variables and parameters of the model (1.1) is given in Table 1.

**Table 1:** **Description of the variables of the vector-host model (**1.1**)**

**Variable**	**Description**
*N*_*H*_(*t*)	Total host population
*S*_*H*_(*t*)	Population of susceptible hosts
*E*_*H*_(*t*)	Population of exposed hosts
*I*_*H*_(*t*)	Population of infected hosts
*R*_*H*_(*t*)	Population of recovered hosts
*N*_*V*_(*t*)	Total vector population
*S*_*V*_(*t*)	Population of susceptible vectors
*E*_*V*_(*t*)	Population of exposed vectors
*I*_*V*_(*t*)	Population of infected vectors

Garba et al. [11] have calculated the basic reproduction number *R*_0 _for their original model. Although, we have made a slight modification to the original model, the basic reproduction number for our model is not significantly different. Hence, for the model (1.1), *R*_0 _is given by

(1.2) $$\begin{array}{l} R_{0}=\sqrt{\frac{C_{\text{HV}}^{2}\Pi_{V}\mu \sigma_{H}\sigma_{V}}{\Pi_{H}\mu_{V}k_{1}k_{2}k_{3}k_{4}}} \end{array}$$

where

$$\begin{array}{ll} k_{1} & =\sigma_{H}+\mu_{H}\;k_{2}=\tau_{H}+\mu_{H}+\delta_{H}\\ k_{3} & =\sigma_{V}+\mu_{V}\;\text{and}\;k_{4}=\mu_{V}+\delta_{V} \end{array}$$

**Figure 10:**
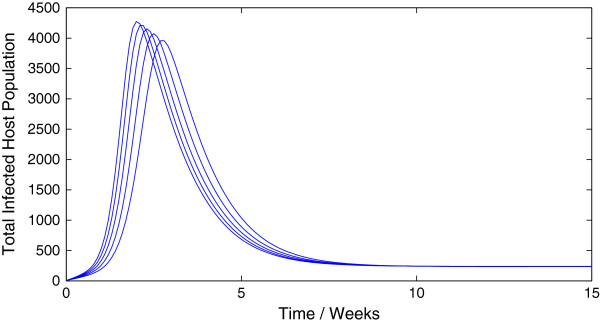
**Sample trajectories of model (**1.1**) with *R***_**0 **_**> 1.**

**Figure 11:**
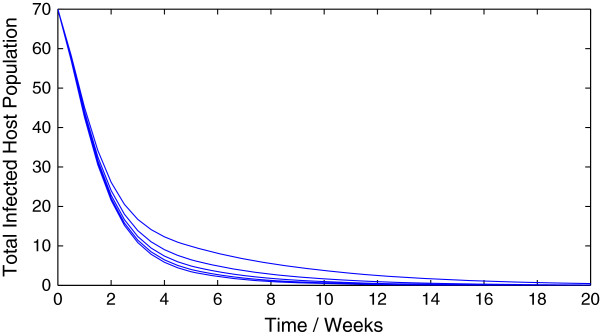
**Sample trajectories of model (**1.1**) with *R***_**0 **_**< 1.**

Representative trajectories of the model (1.1) for different values of *R*_0 _are given in Figure 10 and Figure 11. As expected, a value of *R*_0 _greater than unity leads to an epidemic while a value of *R*_0 _less than unity leads to swift disease elimination.

#### ***Data sources***

**Figure 12:**
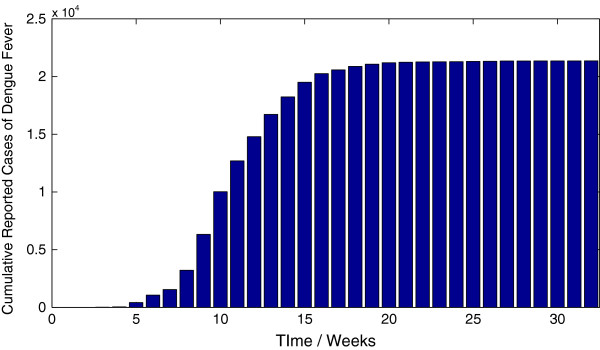
The cumulative number of dengue cases during the 2011 dengue fever epidemic in Pakistan as reported by the Punjab Disaster Management Authority (PDMA).

The epidemic data used in this study was collected by the Punjab Disaster Management Authority (PDMA) during the 2011 Dengue Epidemic in Punjab, Pakistan. The data, displayed in Figure 12, represents the cumulative number of dengue fever cases reported over a 32-week period extending from the 1 ^*st *^of August 2011, to the 20^*th *^of February 2012. The data was collected from a number of public and private hospitals in the major metropolitan centers of Punjab, including Lahore, Faisalabad and Multan. Patients were classified as infected with dengue virus based on the results of laboratory tests for the dengue specific antibodies Immunoglobulin G (IgG) and Immunoglobulin M (IgM). As per the Government of Pakistan’s directives, the laboratory test was available at a subsidized rate in all major hospitals in the capital city of Lahore. It is therefore unlikely that poverty played a serious role in under-reporting of dengue fever cases.

Prior to August 2011, there were three reported cases of dengue fever, all occurring several months before the actual epidemic. This leads us to conclude that these were isolated incidents and were not directly related to the epidemic itself. Furthermore, nearly 87% of all dengue infections were caused by a single serotype (DEN2) of the dengue virus. This justifies our use of a single-strain epidemic model as opposed to dengue models that incorporate the effects of cross-immunity and ADE.

### Estimation schemes

In order to calculate *R*_0_, we require the values of several parameters used in model (1.1). Furthermore, we require knowledge of the initial conditions that will be used to simulate trajectories of the model (1.1).

Estimates for several of the model parameters used in model (1.1) can be obtained from existing studies on Dengue Fever. Table 2 lists these parameters along with reasonable estimates of their values.

**Table 2:** **Description of the parameters of the vector-host model (**1.1**)**

**Parameter**	**Description**	**Value**	**Reference**
*Π*_*H*_	Host recruitment rate	140 week ^-1^	assumed
*Π*_*V*_	Vector recruitment rate	28000 week ^-1^	[12]
$\frac{1}{\mu }$	Host mortality	3494 weeks	[12]
*δ*_*H*_	Host disease-induceddeath rate	0.0035 week ^-1^	[12]
$\frac{1}{\mu_{V}}$	Vector mortality	2 weeks	[12]
*δ*_*V*_	Vector disease-induceddeath rate	negligible week ^-1^	[11]
$\frac{1}{\sigma_{H}}$	Latency period forexposed hosts	1 week [13]	
$\frac{1}{\tau_{H}}$	Recovery time for infectedhosts	1 week	[12]
$\frac{1}{\sigma_{V}}$	Latency period forexposed vectors	$\frac{10}{7}$ weeks	[13]
*C*_*H* *V*_	Effective contact rate	Variable	

The recruitment rate for the susceptible host population depends on the demographics of the urban population that is being considered. This study uses epidemic data collected during the 2011 Dengue Epidemic in Punjab, Pakistan. Therefore, in the absence of concrete estimates, the host recruitment rate has been chosen so as to allow for a realistic steady state host population.

The effective contact rate *C*_*HV*_, which is a measure of the rate at which contact between an infective and a susceptible individual occurs and the probability that such contact will lead to an infection, is extremely difficult to determine directly. Most previous studies have used assumed values for the effective contact rate [11-13]. Thus, it is not possible to directly estimate the basic reproduction number. Consequently, we will adopt an indirect approach, similar to previous studies such as [30] and [18], by first finding the value of the parameter *C*_*HV *_for which the model (1.1) has the best agreement with the epidemic data, and then using the resultant parameter values to estimate *R*_0_.

As mentioned before, for the purpose of simulating model (1.1) we require knowledge of the initial conditions. It is possible to consider the initial conditions (*S*_*H*_(0),*E*_*H*_(0),*I*_*H*_(0),*R*_*H*_(0),*S*_*V*_(0),*E*_*V*_(0),*I*_*V*_(0)) as model parameters along with the effective contact rate *C*_*HV*_ and estimate values for all parameters. Such a technique, however, produces slightly unreliable results. This is explained by the fact that the available epidemic data is restricted to the cumulative number of dengue cases reported, while the optimization schemes that we will employ produces estimates for eight variables. There are thus too many degrees of freedom and the ‘best-fit’ may result in unrealistic estimates for the initial conditions. We will therefore use reasonable estimates for the initial conditions and restrict ourselves to optimizing only the effective contact rate *C*_*HV*_.

Table 3 displays the initial conditions that were therefore chosen.

**Table 3:** **Initial conditions used when applying the OLS and GLS methodology to the vector-host model (**1.1**)**

**Initial condition**	**Value**
*S*_*H*_(0)	1 million
*E*_*H*_(0)	15
*I*_*H*_(0)	3
*R*_*H*_(0)	0
*S*_*V*_(0)	0.1 million
*E*_*V*_(0)	60
*I*_*V*_(0)	20

In the following sections we will employ different methods to estimate the parameter *ω *= *C*_*HV *_by minimizing the difference between the predictions of model (1.1) and the epidemic data.

#### ***Ordinary Least Squares Estimation***

We will first attempt to estimate the effective contact rate by fitting the best trajectory of model (1.1) to the epidemic data using an ordinary least squares (OLS) scheme, implemented using the fminsearch function in the built-in Optimization Toolbox in MATLAB. This will allow us to estimate the parameter *ω* and thus calculate the basic reproduction number *R*_0_.

In order to fit model trajectories with the observed epidemic data, we will assume that all reported cases recovered from the infection after a time lag of two weeks. Thus, the epidemic data, after a lapse of two weeks, represents the total recovered host population. In addition, in order to account for the effect of control measures that were implemented during the actual epidemic, we have assumed that the transmission rate *C*_*HV *_is a function of time *t*. We will assume that the transmission rate was constant up until the point when control measures were implemented, whereupon it changed to a different constant value. An alternative definition of the contact rate is also given in [33]. Thus, mathematically,

(2.1) $$C_{\text{HV}}(t)=\left\{\begin{array}{ll} C_{\text{HV}1} & \;t<t^{*}\\ C_{\text{HV}2} & \;t\geq t^{*} \end{array}\right)$$

where *t*^∗ ^is the time at which control measures were first implemented. For the purpose of this study and in view of no concrete information being available in this regard, we have assumed that *t*^∗ ^= 8 weeks.

For the purpose of this section we shall employ the notation and methodology developed in [30]. Essentially, we will employ inverse problem methodology to obtain estimates of *ω *= *C*_*HV *_by minimizing the difference between the observed weekly cumulative number of recovered host individuals and the model predictions using a ordinary least squares (OLS) criterion. This will be done in the context of an appropriate statistical model.

The ordinary least squares estimation methodology that we will employ is based on model (1.1) as well as an appropriate statistical model for the observation process. Thus, similar to [30], we assume that the model (1.1), together with a ‘true’ value of the parameter *ω*, given by *ω*_0_, perfectly describes the transmission dynamics of the dengue epidemic. Moreover, we assume that the N observations, *Y*_*j*_, given by the epidemic data, are generated by a statistical process. However, the N observations also contain random deviations from the underlying statistical process. Hence, following [30], it is assumed that

(2.2) $$\begin{array}{l} Y_{j}=p(t_{j},\omega_{0})+\varepsilon_{j}\;\text{for}\;j=1,2,3,\ldots ,N \end{array}$$

where *p*(*t*_*j*_, *ω*_0_) denotes where *p*(*t*_*j*_, *ω*_0_) denotes the total number of infected individuals who recover in the time span of week *j* when using the ‘true’ parameter *ω*_0_. Thus, it is assumed that the observed epidemic data is a particular realization of the statistical model (2.2). Under the framework of model (1.1) therefore,

(2.3) $$\begin{array}{l} p(t_{j},\omega_{0})=\overset{t_{j}}{\underset{t_{j-1}}{\int }}\tau_{H}I_{H}(t,\omega_{0})\;\text{for}\;j=1,2,3,\ldots ,N \end{array}$$

where *t*_0 _denotes the time of start of the statistical process and the time of each observation is given by and ordered as *t*_0 _<*t*_1 _<*t*_2 _… .. <*t*_*N*_.

The error terms *ε*_*j*_, are assumed to be independent, identically distributed (i.i.d) random variables, each with zero mean and finite variance. No further assumptions are made regarding the distribution of *ε*_*j*_. In particular, it is not assumed that the distribution is normal. Thus, *E*[ *ε*_*j*_] = 0 and *var *(*ε*_*j*_) = *σ*^2^. Note that, the i.i.d assumption implies that the variance for each error term *ε*_*j *_is identical. We therefore have

$$E\left[Y_{j}\right]=p(t_{j},\omega_{0})\;\text{and}\;\text{var}(Y_{j})=\sigma^{2}\;\text{for}\;j=1,2,3,\ldots ,N$$

For a set of observations ${\left\{Y_{j}\right\}}_{j=1}^{N}$, produced by the statistical model (2.2), we define the statistical estimator *ω*_*OLS *_as

(2.4) $$\begin{array}{l} \omega_{\text{OLS}}(Y)=\text{arg}\underset{\omega \in \Omega }{\text{min}}\overset{j=N}{\underset{j=1}{\sum }}{\left[Y_{j}-p(t_{j},\omega )\right]}^{2}. \end{array}$$

where Ω⊂R is defined as the physically and biologically feasible region for the parameter *ω*. In other words, the statistical estimator is the solution of the following equations

(2.5) $$\begin{array}{l} \overset{j=N}{\underset{j=1}{\sum }}[\;Y_{j}-p(t_{j},\omega )]\frac{\partial }{\partial \omega }p(t_{j},\omega )=0. \end{array}$$

It is clear that the statistical estimator *ω*_*OLS *_is a random variable since each error term *ε*_*j *_is a random variable. Furthermore, the estimator attempts to minimize the distance between the observed weekly cumulative number of recovered host individuals and the predictions of model (1.1). A subsequent detailed description of how to obtain the probability distribution associated with *ω*_*OLS *_is given in [30]. Our goal in the current study will be to obtain the mean of the probability distribution of *ω*_*OLS*_.

#### ***Uncertainty and sensitivity analysis of R***_***0***_

As mentioned previously, the basic reproduction number *R*_0 _for the deterministic model (1.1) is given by (1.2) Thus, the value of *R*_0 _depends on the variables *C*_*HV*_,*Π*_*H*_,*μ*,*σ*_*H*_,*δ*_*H*_,*τ*_*H*_,*Π*_*V*_,*σ*_*V*_,*μ*_*V *_and *δ*_*V*_. While deterministic models implicitly assume that the model parameters are not stochastic in nature, an element of uncertainty is always associated with estimates of these parameters due to factors such as natural variation, errors in measurements and lack of measuring techniques. In general, uncertainty analysis quantifies the degree of confidence in the parameter estimates by producing 95% confidence intervals (CI) which can be interpreted as intervals containing 95% of future estimates when the same assumptions are made and the only noise source is observation error. Additionally, sensitivity analysis identifies critical model parameters and quantifies the impact of each input parameter on the value of an output. In this section, we shall perform uncertainty and sensitivity analysis of the basic reproduction number *R*_0_. A detailed description of the history and methodology of uncertainty and sensitivity analysis is given in [35].

We will use Latin hypercube sampling (LHS) [35] to quantify the uncertainty in and sensitivity of *R*_0 _as a function of the 7 model parameters (*C*_*HV*_,*μ*,*σ*_*H*_,*δ*_*H*_,*τ*_*H*_,*σ*_*V*_,*μ*_*V *_and *δ*_*V*_). It is assumed, following [33], that the recruitment rates *Π*_*V *_and *Π*_*H *_are constants. This will enable us to obtain CI for the value of *R*_0 _that we have estimated in the last section. For the sensitivity analysis, the partial rank correlation coefficient (PRCC) technique [35] will be used to assess the impact of changes in parameter values on the value of *R*_0_. PRCC, which uses rank transformation of the data to reduce the effects of non-linearity, provides a measure of monotonicity after the removal of the linear effects of all but one model parameter. PRCC is therefore a global sensitivity analysis technique. The assumed distributions of the model parameters used in the two analyses are mentioned in Table 4.

**Table 4:** **Assumed probability distributions for the parameters of the model (**1.1**) used in the sensitivity and uncertainty analysis**

**Parameter**	**Mean**	**Variance**	**(Dist)**
*μ*	$\frac{1}{3494}$	$\frac{1}{10^{5}}$	()
*σ*_*H*_	1.4	0.35	()
*δ*_*H*_	0.0035	0.0005	()
*τ*_*H*_	1	0.2	()
*σ*_*V*_	1.4	0.2	()
*μ*_*V*_	0.5	0.1	()
*δ*_*V*_	0.01	0.001	()

#### ***Generalized least squares estimation***

The Ordinary Least Squares Estimation (OLS) scheme we employed in the previous section assumed that the variances associated with the epidemic observations were longitudinally constant and not dependent on the values of the observations. This may not be a realistic assumption especially if the epidemic data is influenced by a source of non-constant systematic error such as under-reporting. Indeed, under-reporting of dengue cases has been well documented in previous studies such as [36] and [37].

If indeed the epidemic data that is being used in the current study is influenced by under-reporting then the assumption of constant variances associated with the observations is not correct since observation errors will now be proportional to the size of the measurement. Hence, we must use a statistical model, which assumes longitudinally non-constant and model dependent variances for the epidemic observations. In this section therefore, we will attempt to estimate the effective contact rate by fitting the best trajectory of model (1.1) to the epidemic data using a generalized least squares (GLS) scheme. An excellent discussion on the use of the OLS and GLS scheme and different statistical models depending on the assumptions regarding the error present in the observation process is given in [38].

Once again we shall employ the notation and methodology developed in [30]. Apart from the assumptions of the statistical model, as before, we will assume that all reported cases recovered from the infection after a time lag of two weeks and that therefore, the epidemic data, after a lapse of two weeks, represents the total recovered host population. Furthermore, we will again assume that the effective contact rate *C*_*HV *_is a function of time. Thus, mathematically, Eq. 2.1 where *t*^∗ ^is the time at which control measures were first implemented. As before, we have assumed that *t*^∗ ^= 8 weeks.

Again, following [30], we will employ inverse problem methodology to obtain estimates of *ω* = *C*_*HV *_by minimizing the difference between the observed weekly cumulative number of recovered host individuals and the model predictions using a generalized least squares (GLS) criterion. This will be done in the context of a statistical model, which assumes that the error in the observation process is directly proportional to the values of the measurement. Hence, it is assumed that

(2.6) $$\begin{array}{l} \;Y_{j}=p(t_{j},\omega_{0})+p(t_{j},\omega_{0})\varepsilon_{j}\;\text{for}\;j=1,2,3,\ldots ,N \end{array}$$

where denotes where *p*(*t*_*j*_,*ω*_0_) denotes the total number of infected individuals who recover in the time span of week *j* using the ‘true’ parameter *ω*_0_. Thus, it is assumed that the observed epidemic data is a particular realization of the statistical model (4.1). Under the framework of model (1.1) therefore,

(2.7) $$\begin{array}{l} p(t_{j},\omega_{0})=\overset{t_{j}}{\underset{t_{j-1}}{\int }}\tau_{H}I_{H}(t,\omega_{0})\;\text{for}\;j=1,2,3,\ldots ,N \end{array}$$

where *t*_0 _denotes the time of start of the statistical process and the time of each observation is given by and ordered as *t*_0 _<*t*_1 _<*t*_2 _….. <*t*_*N*_.

The rest of the analysis is similar to the method outlined in the previous section and follows easily.

### Methods and materials for statistical inference using the direct-transmission model

#### ***The direct transmission epidemic model***

Several existing studies on the transmission dynamics of dengue use a direct transmission SEIR model [13]. The direct transmission models can be obtained using an approximation to vector-host models such as model (1.1). First, it is assumed that the vector force of infection can be approximated by solving for the equilibrium values of the vector population compartments. Furthermore, it is assumed that the susceptible vector population is approximately a linear multiple of the total host population. These two assumptions effectively result in a rescaling of the host effective contact rate *C*_*HV *_of model (1.1) into a direct transmission contact parameter *β*. Using the aforementioned approximation, we formulate a standard incidence, direct transmission SEIR model. More details of the approximation are given in [13].

Mathematically, the direct-transmission model is a follows

(3.1) $$\begin{array}{ll} \frac{\text{dS}(t)}{\text{dt}} & =-\frac{\beta S(t)I(t)}{N(t)}-\mu S(t)\\ \frac{\text{dE}(t)}{\text{dt}} & =\frac{\beta S(t)I(t)}{N(t)}-(\sigma +\mu )E(t)\\ \frac{\text{dI}(t)}{\text{dt}} & =\sigma E(t)-(\tau +\mu +\delta )I(t)\\ \frac{\text{dR}(t)}{\text{dt}} & =\tau I(t)-\mu R(t) \end{array}$$

where

$$\begin{array}{l} N(t)=S(t)+E(t)+I(t)+R(t) \end{array}$$

A description of the variables and parameters of the model (3.1) is given in Table 5 and Tablee 6 respectively.

**Table 5:** **Description of the variables of the direct-transmission model (**3.1**)**

**Variable**	**Description**
*N*(*t*)	Total host population
*S*(*t*)	Population of susceptible hosts
*E*(*t*)	Population of exposed hosts
*I*(*t*)	Population of infected hosts
*R*(*t*)	Population of recovered hosts

**Table 6:** **Description of the parameters of the direct-transmission model (**3.1**)**

**Parameter**	**Description**
$\frac{1}{\mu }$	Host mortality
*δ*	Host disease-induced death rate
$\frac{1}{\sigma }$	Latency period for exposed hosts
$\frac{1}{\tau }$	Recovery time for infected hosts
*β*	Effective contact rate for the direct transmission model

For the direct transmission model (3.1), the basic reproduction number *R*_0 _is defined as the total number of secondary infections produced by introducing a single infected host in a completely susceptible population. Therefore, for model (3.1), the basic reproduction number *R*_0 _is given by:

$$\begin{array}{l} R_{0}=\frac{\beta \sigma }{\left(\tau +\delta +\mu )(\sigma +\mu \right)} \end{array}$$

As discussed previously, the existing literature on dengue fever provides excellent estimates of all the parameters of Model (3.1) with the exception of the contact rate *β*. Hence, our aim will be to estimate the contact rate *β* using statistical inference and thereby estimate the basic reproduction number *R*_0_. The basic methodology used for both the OLS and GLS schemes will be similar to the process outlined in the previous sections.

In order to account for the effect of control measures that were implemented during the actual epidemic, we have assumed that the contact rate *β*(*t*) is a function of time *t*. We will assume that the contact rate was constant up until the point when control measures were implemented, whereupon it changed to a different constant value. Hence, mathematically,

(3.2) $$\beta (t)=\left\{\begin{array}{ll} \beta_{1} & \;t<t^{*}\\ \beta_{2} & \;t\geq t^{*} \end{array}\right)$$

where *t*^∗ ^is the time at which control measures were first implemented. For the purpose of this study and in view of no concrete information being available in this regard, we have assumed that *t*^∗ ^= 6 weeks. We observe that *β*(*t*) is most likely not a continuous function of time *t*. An alternative definition of the transmission rate *β*(*t*), as a continuous function of time, is given in [33].

### The stochastic model and Markov Chain Monte Carlo (MCMC)

In this section we formulate a stochastic, direct-transmission, discrete-time, (S)usceptible, (E)xposed, (I)nfected and (R)ecovered/(R)emoved (SEIR) model for the transmission dynamics of dengue virus. We then use standard Markov chain Monte Carlo (MCMC) methods to perform Bayesian Inference on the epidemic data to obtain estimates of the basic reproduction number *R*_0_. As mentioned in [13], a number of studies exist on the transmission dynamics of dengue that assume direct-transmission. Moreover, a simple approximation can be used to reduce a vector-host model for dengue virus to a direct transmission model (see [13] and the references given therein for more details). The purpose of using a direct-transmission model is to make stochastic inference computationally tractable. For the purpose of this study, we will broadly follow the procedure outlined in [33].

#### Stochastic model formulation

The stochastic SEIR model we will consider is a discrete-time model that has been formulated and discussed previously in [25,33]. Let *S*(*t*),*E*(*t*),*I*(*t*) and *R*(*t*) denote the susceptible host, exposed host, infected host and removed or recovered host populations at time *t* respectively. As is common, our model will assume homogenous mixing of all individuals. Furthermore, consider a time interval (*t*,*t*+*h*], where h denotes the length of time between two observations of the epidemic. In this case therefore, *h* is one week. Now, let *B*(*t*) denote the number of susceptible individuals who have contracted disease, *C*(*t*) the number of exposed individuals who have become infected and *D*(*t*) the number of infected individuals who die or have recovered from the disease during this time interval. For the sake of simplicity, and keeping in view the low mortality rate associated with dengue fever, we will assume that the disease-induced death rate is negligible. Following [33], and in view of the fact that the dynamics of dengue disease take place on a much smaller time scale than the average human life expectancy, we will assume that the total population *N* remains constant. Thus, mathematically the stochastic model is given by

(4.1) $$\begin{array}{ll} S(t+h) & =S(t)-B(t)\\ E(t+h) & =E(t)+B(t)-C(t)\\ I(t+h) & =I(t)+C(t)-D(t)\\ S(t)+E & (t)+I(t)+R(t)=N \end{array}$$

where,

$$\begin{array}{l} B(t)\;∼\text{Bin}(S(t),\lambda (t)),\;C(t)∼\text{Bin}(E(t),k_{\sigma })\;\text{and}\\ B(t)\;∼\text{Bin}(I(t),k_{\tau }) \end{array}$$

are random variables with binomial distributions. The probability of success for these binomial random variables is given by

(4.2) $$\begin{array}{ll} \lambda (t) & =1-\text{exp}\left(\frac{\beta (t)I(t)}{N}h\right)\\ k_{\sigma } & =1-\text{exp}\left(-\sigma h\right)\\ k_{\tau } & =1-\text{exp}(-\tau h) \end{array}$$

Here, $\beta (t),\frac{1}{\sigma }$ and $\frac{1}{\tau }$ are the time dependent transmission rate, the mean latency period and the mean infectious period respectively. Thus, in model (4.1) the transitions from one compartment to another are formulated as an exponentially distributed stochastic movements. The probability that each individual will stay in a specific compartment for a time period *h* is given by *exp*(*Πh*), where *Π* is the compartment specific movement rate. The binomial distributions in (4.2) are then obtained by summing over the individual Bernoulli trials for every individual in the compartment. It is assumed that each trial is independent and identical for every member of the compartment.

Similar to the previous section, we will assume that the contact rate *β*(*t*) is a function of time *t*. Thus, mathematically, Eq. (3.2) where *t*^∗^ is the time at which control measures were first implemented. As before, we have assumed that *t*^∗ ^= 8 weeks.

The stochastic model (4.1) makes use of the parameters *β*(*t*),*σ* and *τ*. Of these parameters, estimates of *σ* and *τ* can be obtained from existing studies on dengue virus and have been given previously. Therefore, our aim will be to estimate the parameter *β*(*t*) using the epidemic data and estimates of initial population sizes. For this purpose, we define the parameter vector, which we will attempt to estimate, as *Θ *= *β*(*t*). Moreover, we define

(4.3) $$\begin{array}{ll} \text{B} & ={\left\{B(t)\right\}}_{t=0}^{t=\tau^{*}}\\ \text{C} & ={\left\{C(t)\right\}}_{t=0}^{t=\tau^{*}}\\ \text{D} & ={\left\{D(t)\right\}}_{t=0}^{t=\tau^{*}} \end{array}$$

where *τ*^∗ ^denotes the time at which observations of the epidemic have finished.

Thus, based on the available epidemic data, we have complete knowledge of both **C **and **D **but no knowledge of **B**. This lack of knowledge will be a major cause of uncertainty in our analysis. Nevertheless, we will attempt to estimate *R*_0 _for both the time period before control measures are implemented and the time period after control measures are implemented using our knowledge of both **C** and **D**.

#### ***Inference methodology***

Based on the definitions given in (4.1) and (4.2), we observe that *B*(*t*),*C*(*t*) and *D*(*t*) are conditionally independent random variables. Thus, the likelihood function for the data set {**B**,**C**,**D**} is given by

(4.4) $$\begin{array}{l} L(\text{B},\text{C},\text{D}|\Theta )=\overset{t=\tau^{*}}{\underset{t=0}{\prod }}f_{1}(B(t)|.)f_{2}(C(t)|.)f_{3}(D(t)|.) \end{array}$$

where *f*_1_,*f*_2 _and *f*_3 _are the binomial transition probabilities given in (4.1) and (4.2), conditioned on *Θ *and all the epidemic data represented by **B**, **C** and **D **up until time *t*. Therefore, the maximum likelihood estimator for the parameter vector *Θ*, and by extension for *β*(*t*) and *R*_0 _can be obtained by maximizing the expression in (4.4).

According to model (4.1), the time series for *S*(*t*),*E*(*t*),*I*(*t*) and *R*(*t*) can be obtained using *B*(*t*),*C*(*t*) and *D*(*t*). Unfortunately as mentioned previously, *B*(*t*) is unknown since the process of infection is not observed. Hence, we must also impute the values of *B*(*t*). These values can then be used to construct the time series for *S*(*t*) and *E*(*t*).

Since, the likelihood function for **B**,**C** and **D**, denoted as *L*(**B**,**C**,**D**|*Θ*), is given by (4.4), we can use Bayes’ Theorem to obtain, up to a constant, the required posterior distribution that we wish to sample from. This is given by

(4.5) $$\begin{array}{l} L(\Theta ,\text{B}|\text{C},\text{D})\propto L(\text{B},\text{C},\text{D}|\Theta )\pi (\Theta ) \end{array}$$

where *π*(*Θ*) is the prior distribution. Thus, our MCMC algorithm will sample from the conditional probability distributions *π*(*Θ*|**B**,**C**,**D**) and *π*(**B**|*Θ*,**C**,**D**) to produce samples from the required distribution *π*(*Θ*,**B**|**C**,**D**). In short, our general algorithm will proceed as follows 

• Initialize the set **B** using any appropriate initial vector.

• Since, **C** and **D** are known, construct the time series for *S*(*t*),*E*(*t*),*I*(*t*) and *R*(*t*).

• Initialize the parameter vector *Θ*.

• Update **B** using the conditional distribution *π*(**B**|*Θ*,**C**,**D**).

• Reconstruct the new time series for *S*(*t*),*E*(*t*),*I*(*t*) and *R*(*t*).

• Update *Θ* using the conditional distribution *π*(*Θ*|**B**,**C**,**D**).

• Repeat steps 4-6 until the Markov chain has converged and subsequently, the required samples have been obtained.

To sample from *π*(**B**|*Θ*,**C**,**D**) one can use the conditional binomial distribution for **B**, making sure that the choice is consistent with the final size and length of the epidemic. This is however computationally very inefficient as most of the draws would be rejected due to the consistency condition. To avoid this issue we condition the proposal on the observed extinction time, following the method described in [33] for computationally efficient sampling. *π*(*Θ*|**B**,**C**,**D**) is updated using a random walk proposal.

#### ***Inference from the observed dengue data***

An important question that arises at this point pertains to the meaning and significance of the basic reproduction number *R*_0_ for the stochastic SEIR model. As mentioned previously and as discussed in detail in [31], the basic reproduction number *R*_0_ for the deterministic SEIR model is essentially a threshold quantity which determines the possibility of an outbreak of the disease. Thus, for the deterministic SEIR model, if *R*_0_ is less than unity there is no epidemic while if *R*_0_ is greater than unity there will be a disease epidemic.

Unfortunately, the threshold dynamics of the stochastic SEIR model are not the same. It can be proven that in contrast to the deterministic model, the stochastic SEIR model predicts disease extinction regardless of the value of *R*_0_. This results in difficulty regarding the interpretation of *R*_0 _as a threshold quantity. Therefore, it is tempting to ask the question: what is the importance of *R*_0 _in the stochastic SEIR model? An answer to this question may be conjectured (but not proven) by referring to [39]. It is proven in [39] that for the stochastic SI model, on average no epidemic will occur if *R*_0 _< 1, while for *R*_0 _> 1 there is a finite probability that an endemic quasi-equilibrium will develop. We conjecture that this result also holds true for the stochastic SEIR model and that it can therefore be used to explain the significance of *R*_0_ as a threshold quantity for the stochastic SEIR model.

Using the MCMC algorithm discussed in the previous section, we estimate the transmission rate *β*(*t*) and hence the basic reproduction number *R*_0_, both for the time period before control measures are implemented and the time period after the control measures are implemented. We have taken *t*^∗ ^= 8 weeks and the initial population sizes to be the same as in the case of inference from the deterministic model using the GLS and OLS schemes. Furthermore, we have taken an uniform prior distribution for the parameter vector *Θ*. We observe that the results of the MCMC algorithm, displayed in Table 7, are in close agreement with the corresponding results obtained from application of the OLS and GLS schemes to the direct-transmission deterministic model.

**Table 7:** **Posterior mean of the contact rate and basic reproduction number for the Stochastic direct-transmission model (**4.1**)**

**Parameter**	**Posterior mean**
*β*_1_	3.0650 week ^-1^
*β*_2_	0.6318 week ^-1^
*R*_0_ before control measures	3.0528
*R*_0_ after control measures	0.6293

## Competing interests

The authors declare that they have no competing interests.

## Authors’ contributions

MI and AK gave initial input and directed the research. MH implemented the algorithms, carried out the programming and drafted the manuscript. AK interpreted the results and edited the manuscript. All authors read and approved the final manuscript.

## Supplementary Material

Additional file 1 Multilingual abstracts in the six official working languages of the United Nations. Click here for file

## References

[B1] Ranjit S Kissoon N **Dengue hemorrhagic fever and shock syndromes** Pediatr Crit Care Med 2011 12 90 100 10.1097/PCC.0b013e3181e911a7 20639791

[B2] **World Health Organization: dengue and severe dengue fact sheet** 2012 [http://www.who.int/mediacentre/factsheets/fs117/en/]

[B3] Gubler DJ **Dengue and dengue hemorrhagic fever** Clin Microbiol Rev 1998 11 3 480 496 9665979 10.1128/cmr.11.3.480 PMC88892

[B4] Halstead S Nimmannitya S Cohen S **Observations related to pathogenesis of dengue hemorrhagic fever. IV. Relation of disease severity to antibody response and virus recovered** Yale J Biol Med 1970 42 5 311 322 5419206 PMC2591704

[B5] Kautner I Robinson MJ Kuhnle U **Dengue virus infection: epidemiology, pathogenesis, clinical presentation, diagnosis, and prevention** J Pediatr 1997 131 4 516 524 10.1016/S0022-3476(97)70054-4 9386651

[B6] Shekhar C **Deadly dengue: new vaccines promise to tackle this escalating global menace** Chem Biol 2007 14 8 871 872 10.1016/j.chembiol.2007.08.004 17719483

[B7] Holmes EC Twiddy SS **The origin, emergence and evolutionary genetics of dengue virus** Infect Genet Evol 2003 3 19 28 10.1016/S1567-1348(03)00004-2 12797969

[B8] Whitehorn J Farrar J **Dengue** Br Med Bull 2010 95 161 173 10.1093/bmb/ldq019 20616106

[B9] Gubler D Kuno G Dengue and Dengue Hemorrhagic Fever 1997 London: CAB INTERNATIONAL

[B10] Kawaguchi I Sasaki A Boots M **Why are dengue virus serotypes so distantly related? Enhancement and limiting serotype similarity between dengue virus strains** Proc R Soc Lond B Biol Sci 2003 270 1530 2241 2247 10.1098/rspb.2003.2440 PMC1691498 14613610

[B11] Garba SM Gumel AB **Abu Bakar MR: Backward bifurcations in dengue transmission dynamics** Math Biosci 2008 215 11 25 10.1016/j.mbs.2008.05.002 18573507

[B12] Garba S Gumel A **Effect of cross-immunity on the transmission dynamics of two strains of dengue** Int J Comput Math 2010 87 10 2361 2384 10.1080/00207160802660608

[B13] Wearing HJ Rohani P **Ecological and immunological determinants of dengue epidemics** Proc Natl Acad Sci 2006 103 31 11802 11807 10.1073/pnas.0602960103 16868086 PMC1544250

[B14] Esteva L Vargas C **Coexistence of different serotypes of dengue virus** J Math Biol 2003 46 31 47 10.1007/s00285-002-0168-4 12525934

[B15] Ferguson N Anderson R Gupta S **The effect of antibody-dependent enhancement on the transmission dynamics and persistence of multiple-strain pathogens** Proc Natl Acad Sci 1999 96 2 790 794 10.1073/pnas.96.2.790 9892712 PMC15215

[B16] Esteva L Vargas C **A model for dengue disease with variable human population** J Math Biol 1999 38 3 220 240 10.1007/s002850050147 10220925

[B17] Esteva L Vargas C **Analysis of a dengue disease transmission model** Math Biosci 1998 150 2 131 151 10.1016/S0025-5564(98)10003-2 9656647

[B18] Chowell G Diaz-Dueñas P Miller J Alcazar-Velazco A Hyman J Fenimore P Castillo-Chavez C **Estimation of the reproduction number of dengue fever from spatial epidemic data** Math Biosci 2007 208 2 571 589 10.1016/j.mbs.2006.11.011 17303188

[B19] Allen LJ Brauer F, Driessche P, Wu J **An introduction to stochastic epidemicmodels** Mathematical Epidemiology, Volume 1945 of Lecture Notes in Mathematics 2008 Springer Berlin Heidelberg, 14197 Berlin Germany 81 130

[B20] Keeling MJ Ross JV **On methods for studying stochastic disease dynamics** J R Soc Interface 2008 5 19 171 181 10.1098/rsif.2007.1106 17638650 PMC2705976

[B21] Bailey NT **A simple stochastic epidemic** Biometrika 1950 37 193 202 10.1093/biomet/37.3-4.193 14801045

[B22] Allen LJ Flores DA Ratnayake RK Herbold JR **Discrete-time deterministic and stochastic models for the spread of rabies** Appl Math Comput 2002 132 2 271 292

[B23] Weiss GH Dishon M **On the asymptotic behavior of the stochastic and deterministic models of an epidemic** Math Biosci 1971 11 3 261 265

[B24] Tuite AR Tien J Eisenberg M Earn DJ Ma J Fisman DN **Cholera epidemic in Haiti, 2010: using a transmission model to explain spatial spread of disease and identify optimal control interventions** Ann Intern Med 2011 154 9 593 601 10.7326/0003-4819-154-9-201105030-00334 21383314

[B25] Allen L Driessche P **Stochastic epidemic models with a backward bifurcation** Math Biosci Eng 2006 3 3 445 20210373 10.3934/mbe.2006.3.445

[B26] de Souza DR Tomé T Pinho ST Barreto FR de Oliveira MJ **Stochastic dynamics of dengue epidemics** Phys Rev E 2013 87 012709 10.1103/PhysRevE.87.012709 23410361

[B27] Spencer S Stochastic epidemic models for emerging diseases. PhD thesis 2008 University of Nottingham

[B28] Allen LJ An Introduction to Stochastic Processes with Applications to Biology 2003 New Jersey: Pearson Education

[B29] Allen LJ Burgin AM **Comparison of deterministic and stochastic SIS and SIR models in discrete time** Math Biosci 2000 163 1 33 10.1016/S0025-5564(99)00047-4 10652843

[B30] Cintrón-Arias A Castillo-Chávez C Bettencourt LM Lloyd AL Banks H **The estimation of the effective reproductive number from disease outbreak data** Math Biosci Eng 2009 6 2 261 282 19364152 10.3934/mbe.2009.6.261

[B31] Van den Driessche P Watmough J **Reproduction numbers and sub-threshold endemic equilibria for compartmental models of disease transmission** Math Biosci 2002 180 29 48 10.1016/S0025-5564(02)00108-6 12387915

[B32] Chowell G Hengartner N Castillo-Chavez C Fenimore F Hyman J **The basic reproductive number of Ebola and effects of public health measures: the cases of Congo and Uganda** J Theor Biol 2004 229 119 126 10.1016/j.jtbi.2004.03.006 15178190

[B33] Lekone PE Finkenstädt BF **Statistical inference in a stochastic epidemic SEIR model with control intervention: Ebola as a case study** Biometrics 2006 62 4 1170 1177 10.1111/j.1541-0420.2006.00609.x 17156292

[B34] O’Neill P Roberts GO **Bayesian inference for partially observed stochastic epidemics** J R Statisitcal Soc A 1999 162 121 129 10.1111/1467-985X.00125

[B35] Sanchez MA Blower SM **Uncertainty and sensitivity analysis of the basic reproductive rate: tuberculosis as an example** Am J Epidemiol 1997 145 12 1127 1137 10.1093/oxfordjournals.aje.a009076 9199543

[B36] Suaya JA Shepard DS Beatty ME **Dengue: burden of disease and costs of illness** TDR. Report of the Scientific Working Group Meeting on Dengue 2006 Geneva Switzerland: World Health Organization 35 49

[B37] Beatty ME Beutels P Meltzer MI Shepard DS Hombach J Hutubessy R Dessis D Coudeville L Dervaux B Wichmann O Margolis HS Kuritsky JN **Health economics of dengue: a systematic literature review and expert panel’s assessment** Am J Trop Med Hyg 2011 84 3 473 488 10.4269/ajtmh.2011.10-0521 21363989 PMC3042827

[B38] Banks HT Davidian M Jr Samuels JR Sutton KL An Inverse Problem Statistical Methodology Summary 2009 3994 AK Houten Netherland

[B39] Jacquez JA O’Neill P **Reproduction numbers and thresholds in stochastic epidemic models I. Homogeneous populations** Math Biosci 1991 107 2 161 186 10.1016/0025-5564(91)90003-2 1806112

