# Supplementary material for: Estimating the basic reproduction number for single-strain dengue fever epidemics
Source: Infect Dis Poverty. 2014 Apr 7;3:12. doi: 10.1186/2049-9957-3-12 (PMC4021574; doi:10.1186/2049-9957-3-12)
Supplement: Additional file 1 — Multilingual abstracts in the six official working languages of the United Nations. [file 2049-9957-3-12-S1.pdf]

Translation of the abstract into the six official working languages of the United Nations

تقدير عدد التكاثر القاعدي لأوبئة حمى الضنك ذات السلالة الواحدة

آدم خان و محمد حسن ومدصر عمران

ملخص

معلومات أساسية: برز في الآونة الأخيرة مرض الضنك، وهو مرض معدي ويوجد في المناطق المدارية، باعتباره من أهم الأمراض الفيروسية التي ينقلها البعوض في العالم. سنقوم بتحليل وباء حمى الضنك لسنة 2011 بباكستان بصفة رجعية وذلك بهدف تقييم قدرة المرض على الانتقال. لقد تحصلنا على تقدير R_0 كعدد قاعدي للتكاثر من خلال البيانات الوبائية وباعتماد منهجيات مختلفة طبقت على نماذج مختلفة من الوباء بهدف تقييم مدى صلابة تقديراتنا.

الطرق: قدرنا أولا البرامترات النموذجية من خلال تكييف نموذج المضيف الناقل من ODE القطعي لديناميكيات انتقال الضنك آحادي السلالة مع المعطيات الوبائية وذلك باستعمال مخطط المربعات الصغرى العادية القاعدية (OLS) وكذلك مخطط المربعات الصغرى المعممة (GLS) . علاوة على ذلك فإننا نجري نفس التحليل للنقل المباشر لنموذج ODE، مما يمكننا من مقارنة نتائجنا عبر نتائج مختلفة. إضافة إلى ذلك فإننا نصوغ نموذج عشوائي للانتقال المباشر لديناميكيات انتقال الضنك والحصول على تقديرات المَعلَمَات للنموذج العشوائي باستعمال طرق سلسلة ماركوف مونتي كارلو (MCMC).

النتائج: في كل الحالات التي تمت دراستها فإن التقديرات حول عدد التكاثر القاعدي R_0 **هو بداية أكبر من الوحدة مما يؤدي إلى تفشي الوباء** . **لكن** تدابير الرقابة التي تم تنفيذها عدة أسابيع منذ تفشي الوباء خفضت بنجاح من R_0 **إلى أقل من وحدة مما أدى إلى القضاء على المرض.** أبعد من ذلك فإن تقديرنا لقيمة R_0 ما قبل السيطرة على المرض وباعتماد منهجيات ونماذج مختلفتين لهي متشابهة جدا. لكن توجد فروقات كبيرة بين تقديراتنا حول القيمة عند الرقابة اللاحقة للعدد القاعدي للتكاثر عبر نموذجين مختلفين.

الاستنتاج: لقد تحصلنا كاستنتاج على تقديرات قوية حول قيمة عدد التكاثر القاعدي R_0 **المرتبطة بوباء حمى الضنك لسنة 2011** في باكستان قبل تطبيق اجراءات الصحة العمومية. أبعد من ذلك، لقد أظهرنا أن تقديراتنا حول قيمة R_0 **عند الرقابة اللاحقة و عبر المنهجيات المختلفة لهي متشابهة جدا. لكن، هناك كذلك فروقات هامة بين تقديراتنا لقيمة R_0 عند الرقابة اللاحقة عبر نموذجين مختلفين.**

Translated from English version into Arabic by Malika2012, through

TRANSLATORS WITHOUT BORDERS

基于基本繁殖数估计单型登革热的流行

Adnan Khan, Muhammad Hassan, and Mudassar Imran

摘要

引言：作为热带传染病的一种，登革热目前已经成为全球最具威胁的蚊媒传播疾病之一。本研究对巴基斯坦2011年登革热流行数据进行回顾性分析，以评估其流行程度。通过不同方法学和流行病学模型获得基本繁殖数R_0，以评估本研究结果的准确性。

方法： 本研究通过应用普通最小二乘法和广义最小二乘法确定估计参数，并校正单型登革热传播动力学模型（ODE）。此外，通过直接传播ODE模型进一步验证本研究结果在不同模型间的效果。进一步通过建立登革热传播动力学的直接传播随机模型，通过马尔科夫-蒙特卡洛（MCMC）方法获得模型的参数估计值。

结果：在所有的研究病例中，最开始的病例基本繁殖数估计值 R_0 高于总体值，因此导致了登革热的爆发流行。然而，爆发之后随着防治措施的有效实施，其 R_0 开始减少并小于总体值，进而达到登革热的消除。虽然本研究对控制前 R_0 估计值在不同方法学和模型验证结果中基本一致，但是在控制后结两种模型中的结果有显著性的差异。

结论：本研究得到 2011 年巴基斯坦登革热流行的基本繁殖数 R_0。该值在疾病控制前在不同的模型中得到一致的结果，但控制后在两种模型中的结果有显著性差异。

Translated from English version into Chinese by Zhang Shao-sen, through

CHINESE CENTER FOR DISEASE CONTROL AND PREVENTION
NATIONAL INSTITUTE FOR PARASITIC DISEASES

Edited by Prof Zhou Xiao-nong (National Institute of Parasitic Diseases, Chinese Center for Disease Control and Prevention)

Estimation du taux de reproduction de base d'une seule souche du virus de la dengue

Adnan Khan, Muhammad Hassan, et Mudassar Imran

Résumé

Contexte : la dengue est une maladie infectieuse tropicale qui a récemment été identifiée comme étant une des maladies virales les plus importantes transmises par les moustiques au monde. Nous avons effectué une analyse rétrospective de l'épidémie de dengue qu'a connue le Pakistan en 2011 afin d'évaluer la transmissibilité de la maladie. Nous avons obtenu une estimation du taux de reproduction de base R_0 des données épidémiques en utilisant différentes méthodologies que nous avons appliquées aux différents modèles épidémiques afin d'évaluer la justesse de nos estimations.

Méthodes: tout d'abord, nous avons estimé les paramètres du modèle par adaptation d'un modèle vecteur-hôte déterministe d'équation différentielle ordinaire (EDO) pour la dynamique de transmission d'une seule souche du virus de la dengue aux données épidémiques, en utilisant à la fois la méthode des moindres carrés ordinaires (MCO) et celle des moindres carrés généralisés (MCG). De plus, nous avons effectué la même analyse pour un modèle EDO de transmission directe ce qui nous a permis de comparer nos résultats entre les différents modèles. En outre, nous avons élaboré un modèle stochastique de transmission directe pour la dynamique de transmission de la dengue et avons obtenu une estimation des paramètres pour le modèle stochastique en utilisant les méthodes de Monte Carlo par chaîne de Markov (MCCM).

Résultats : pour chacun des cas étudiés, l'estimation du taux de reproduction de base R_0 est initialement supérieur à l'unité, menant à une poussée épidémique. Toutefois, les mesures de protection mises en place plusieurs semaines après la première poussée épidémique ont permis de réduire le taux R_0 à moins d'une unité, résultant donc à une élimination de la maladie. De plus, nos estimations de la valeur du taux R_0 avant les mesures de protection, effectuées à l'aide des différentes méthodologies et des différents modèles, sont très similaires. Cependant, il existe également des différences considérables dans nos estimations de la valeur du taux de reproduction de base après les mesures de protection entre les deux différents modèles.

Conclusion : pour conclure, nous avons obtenu des estimations justes pour la valeur

du taux de reproduction de base R_0 liée à l'épidémie de dengue au Pakistan avant la mise en œuvre des mesures de protection de la santé publique. En outre, nous avons démontré que nos estimations de la valeur du taux R_0 avant les mesures de protection sont très similaires, quelle que soit la méthode utilisée. Toutefois, il existe également des différences considérables dans nos estimations de la valeur du taux R_0 après les mesures de protection entre les deux différents modèles.

Translated from English version into French by CelineG, through

TRANSLATORS
WITHOUT BORDERS

Расчет базового репродуктивного числа для одноштаммовой эпидемии лихорадки денге

Adnan Khan, Muhammad Hassan и Mudassar Imran

Реферат

Исходные сведения. Денге, тропическая инфекционная болезнь, в последнее время стала одной из наиболее значимых в мире вирусных инфекций, переносимых комарами. В целях оценки трансмиссивности данной инфекции мы провели ретроспективный анализ эпидемии лихорадки денге, имевшей место в Пакистане в 2011 году. Мы произвели расчет базового репродуктивного числа (R_0) на основе эпидемических данных и использовали различные методы анализа в применении к различным эпидемическим моделям для оценки робастности наших расчетов.

Методы. Вначале мы определили параметры модели, применив к эпидемическим данным детерминированную ОДУ-модель "переносчик–хозяин" для расчета динамики передачи инфекции. При этом были использованы как обычный (OLS), так и обобщенный метод наименьших квадратов (GLS). Помимо этого, мы провели такой же анализ с применением ОДУ-модели прямой передачи инфекции, что позволило нам сравнить результаты, полученные разными методами. Наконец, мы сформулировали стохастическую модель прямой передачи для динамики передачи денге, определив параметры для стохастической модели с использованием анализ Монте-Карло с цепями Маркова.

Результаты. В каждом из проведенных анализов расчетное значение базового репродуктивного числа R_0 на начальном этапе превышало единицу, что вело к развитию эпидемической вспышки. Однако в результате проведенных мер контроля через несколько недель после возникновения вспышки R_0 снизилось до значений менее единицы, таким образом обусловив прекращение распространения болезни. Расчеты значения R_0 по состоянию до начала применения мер контроля дали весьма сходные результаты для всех методов анализа. Вместе с тем, полученные значения базового репродуктивного числа по состоянию после применения мер контроля значительно различались между двумя моделями.

Вывод. Мы получили робастные расчетные результаты значения базового

репродуктивного числа R_0 для эпидемии лихорадки денге 2011 года в Пакистане по состоянию до принятия медико-санитарных мер контроля. Расчеты постконтрольного значения R_0 дали весьма сходные результаты для различных методов анализа. Вместе с тем, расчетные постконтрольные значения R_0 значительно различались между двумя моделями.

Translated from English version into Russian by Vladimir2012, through

TRANSLATORS
WITHOUT BORDERS

Cálculo de la tasa de reproducción básica de la epidemia del dengue de una sola cepa

Adnan Khan, Muhammad Hassan y Mudassar Imran

Resumen

Antecedentes: El dengue, una enfermedad tropical infecciosa, se ha convertido recientemente en una de las principales enfermedades virales portadas por mosquitos en todo el mundo. Realizamos un análisis retrospectivo de la epidemia del dengue de 2011 en Paquistán con la finalidad de evaluar la transmisibilidad de la enfermedad. Obtenemos cálculos de la tasa básica de reproducción R_0 a partir de los datos de la epidemia usando distintas metodologías aplicadas a diferentes modelos epidémicos para evaluar la solidez de nuestros cálculos.

Métodos: Primero, calculamos los parámetros modelo adecuando un modelo determinístico de vector-hospedador de EDO (Ecuación Diferencial Ordinaria) para las dinámicas de transmisión del dengue de una sola cepa a los datos de la epidemia, usando tanto un esquema de mínimos cuadrados ordinarios (MCO) como un esquema de mínimos cuadrados generalizados (MCG). Por otra parte, realizamos el mismo análisis para un modelo de EDO de transmisión directa, que nos permite comparar nuestros resultados a través de los distintos modelos. Además, formulamos un modelo estocástico de transmisión directa para las dinámicas de transmisión del dengue y obtenemos cálculos de parámetro para el modelo estocástico usando los métodos de Monte Carlo vía Cadena de Márkov (MCMC).

Resultados: En cada uno de los casos considerados, el cálculo de la tasa básica de reproducción R_0 es inicialmente mayor que la unidad, lo que conduce a un brote epidémico. Sin embargo, las medidas de control implementadas varias semanas después del brote inicial reducen satisfactoriamente la R_0 a menos de la unidad, de este modo se consigue la eliminación de la enfermedad. Además, nuestros cálculos para el valor de precontrol de la R_0, tanto a través de las distintas metodologías como de los distintos modelos, son muy similares. Sin embargo, también existen diferencias importantes en nuestros cálculos del valor de postcontrol de la tasa de reproducción básica a través de los dos distintos modelos.

Conclusión: En conclusión, hemos obtenido cálculos sólidos para el valor de la tasa de reproducción básica R_0 asociados con la epidemia del dengue de 2011 en Paquistán antes de la implementación de las medidas de control de la salud pública. Asimismo, hemos demostrado que nuestros cálculos del valor de postcontrol de la R_0 son muy similares en las distintas metodologías. Sin embargo, también existen diferencias importantes en nuestros cálculos del valor de postcontrol de la R_0 a través de los dos distintos modelos.

Translated from English version into Spanish by Ibeth Ibarra Anavia Ibs_ia, through

TRANSLATORS
WITHOUT BORDERS
